# Chemical-free Reactive Melt Processing of Biosourced Poly(butylene-succinate-adipate) for Improved Mechanical Properties and Recyclability

**DOI:** 10.1021/acsapm.4c00514

**Published:** 2024-05-13

**Authors:** Michele Gammino, Claudio Gioia, Andrea Maio, Roberto Scaffaro, Giada Lo Re

**Affiliations:** † Department of Engineering, University of Palermo, Viale delle Scienze, Ed. 6, 90128 Palermo, Italy; ‡ Department of Physics, University of Trento, via Sommarive 14, Povo, 38123 Trento, Italy; § Department of Industrial and Materials Science, Chalmers University of Technology, Rannvagen 2A, 41258 Gothenburg, Sweden; ∥ Wallenberg Wood Science Centre, Kemigården 4, 41258 Gothenburg, Sweden

**Keywords:** PBSA, biodegradable polymer, green reactive processing, chemical modification, recycling, mechanical properties, NMR, Biosourced polymer

## Abstract

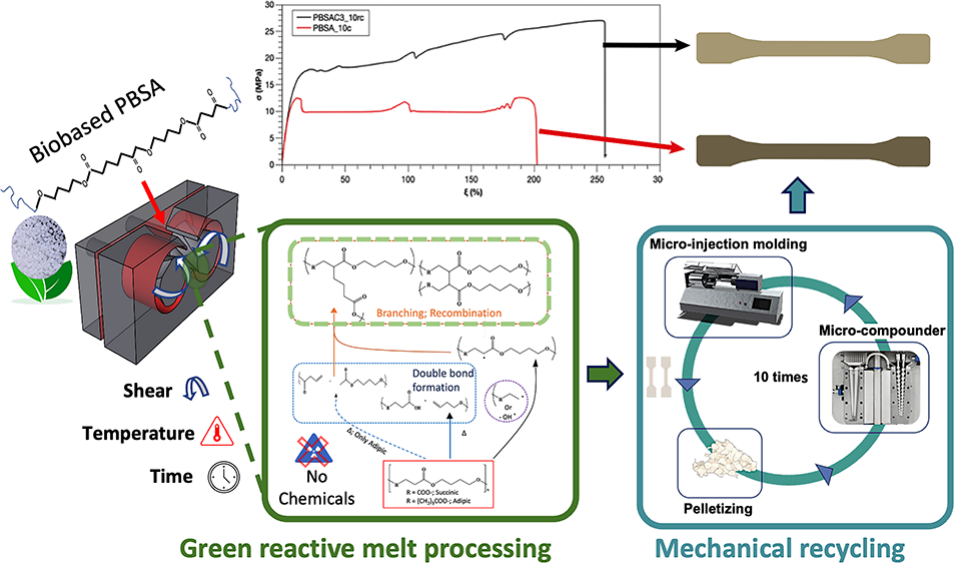

Biosourced and biodegradable polyesters like poly(butylene succinate-*co*-butylene adipate) (PBSA) are gaining traction as promising alternatives to oil-based thermoplastics for single-use applications. However, the mechanical and rheological properties of PBSA are affected by its thermomechanical sensitivity during its melt processing, also hindering PBSA mechanical recycling. Traditional reactive melt processing (RP) methods use chemical additives to counteract these drawbacks, compromising sustainability. This study proposes a green reactive method during melt compounding for PBSA based on a comprehensive understanding of its thermomechanical degradative behavior. Under the hypothesis that controlled degradative paths during melt processing can promote branching/recombination reactions without the addition of chemical additives, we aim to enhance PBSA rheological and mechanical performance. An in-depth investigation of the in-line rheological behavior of PBSA was conducted using an internal batch mixer, exploring parameters such as temperature, screw rotation speed, and residence time. Their influence on PBSA chain scissions, branching/recombination, and cross-linking reactions were evaluated to identify optimal conditions for effective RP. Results demonstrate that specific processing conditions, for example, twelve minutes processing time, 200 °C temperature, and 60 rpm screw rotation speed, promote the formation of the long chain branched structure in PBSA. These structural changes resulted in a notable enhancement of the reacted PBSA rheological and mechanical properties, exhibiting a 23% increase in elastic modulus, a 50% increase in yield strength, and an 80% increase in tensile strength. The RP strategy also improved PBSA mechanical recycling, thus making it a potential replacement for low-density polyethylene (LDPE). Ultimately, this study showcases how finely controlling the thermomechanical degradation during reactive melt processing can improve the material’s properties, enabling reliable mechanical recycling, which can serve as a green approach for other biodegradable polymers.

## Introduction

Poor and ineffective management of nonbiodegradable plastic waste leads to environmental accumulation of micro- and nanoplastics.^1−5^ The development of Biosourced polyesters with biodegradable characteristics, such as polylactic acid (PLA),^6^ poly(hydroxyl alkanoates) (PHAs),^2^ and more recently poly(butylene succinate-*co*-butylene adipate) copolymer (PBSA)^6−11^ contribute to counteract this treat for the planet and human health. Despite the obvious environmental advantage of this class of materials, one of the main challenges hampering their commercial applications concerns their sensitivity to thermomechanical processing. The melt processing at relatively elevated temperatures of biodegradable polyesters during their manufacturing and their mechanical recycling leads to their thermomechanical degradation.^12^ The degradative pathway of such phenomena occurs via radical or nonradical chain splitting reactions^13^ as observed for poly(butylene-*co*-adipate terephthalate) (PBAT),^14^ and poly(butylene succinate) PBS,^10^ poly(ethylene terephthalate) PET,^15^ PLA,^16,17^ and even for polyolefin, such as polypropylene (PP).^18^ These degradation processes induce a decrease in the polymer molar mass, an increased opacity, and the deterioration of the mechanical performance. PBSA is currently gaining traction in the market due to its mechanical properties, similar to those of low-density polyethylene^19^ but with higher deformability than PHAs and PLA.^17,20,21^ Promoting an improvement of PBSA thermomechanical sensitivity as well as its rheological and mechanical properties would further enable its application in the market.^22^ To the best of our knowledge, no recent scientific publication focused on finely exploring the thermomechanical degradative behavior of PBSA; however, due to its similarity with poly(butylene succinate) (PBS), several assumptions can be drawn. Resch-Fauster et al.^23^ have investigated the thermomechanical degradation of PBS after repeated melt processing in an intermeshing corotating twin screw extruder as a simulation of PBS mechanical recycling. The study reported the progressively increased melt flow index (MFI) as a function of the extrusion cycle of PBS and correlated the MFI increase to a decrease in the PBS molar mass (*M*_w_) up to 23% after 7 extrusion cycles at 230 °C. The effect of thermomechanical degradation on PBS viscosity and molar mass induced by reprocessing up to 5 cycles in a similar TSE at different temperatures (from 190 to 210 °C) has also been studied by Georgousopoulou et al.^24^ The work focuses on the use of antioxidants to limit the structural change of PBS during its extrusion and melt reprocessing, that is, mechanical recycling. By studying the PBS intrinsic viscosity and molar mass evolutions as a function of extrusion cycles, the authors propose that the degradative behavior of PBS is governed by thermo-oxidative chain scission combined with branching/recombination reactions. Overall, after an initial decrease, an increase in PBS intrinsic viscosity and molar mass was observed by increasing the processing cycles, a trend that could be anticipated by melt processing the polymer at higher temperatures. Rizzarelli and Carroccio^25,26^ proposed a thermo-oxidative degradation mechanism in which the hydroperoxyl intermediate (ROOH) plays a key role. They proposed three alternative pathways, characterized by different induction times and oligomeric species: (i) hydroxyl ester (ROH), (ii) peroxyl (ROO**·**), (iii) alkyl (R**·**), and alkoxylic radicals (RO**·**). Similar degradative pathways, although induced by gamma irradiation, have been reported for PBSA by Pérez-Valdez.^27^ In this study, a significant decrease of the PBSA physical properties due to a prevalent chain scission compared to branching/recombination reactions was demonstrated as a consequence of radical formation induced by γ radiation and strongly dependent on the radiation dose. Similar processes could result from the thermo-mechanical stress induced during reactive melt processes (RP), an advanced technique using melt processing equipment for carrying out different chemical reactions of components during their manufacturing.^28^ Commonly, RP does not require organic solvents, resulting in a green, single-step, cost-effective process with easy upscaling for industrial uptake.^29^ Several approaches to RP, induced by chain extenders, cross-linking agents, branching agents or a combination of them, have been studied to counteract the drawbacks of thermomechanical degradation during melt processing to enhance chemical and physical, such as rheological and mechanical, properties of different biodegradable polyesters.^27^ A possible drawback of RP consists of the common involvement of chemical additives and/or functionalizing agents, which may affect the overall sustainability and the end-of-life of the material. Under the rationale of intentionally inducing branching/recombination reactions in commercial PBSA to enhance its physical properties, we propose a reactive melt processing approach grounded on a deeper understanding of PBSA thermomechanical degradative behavior. We hypothesize that the control of the PBSA degradative paths during its melt processing can be exploited to promote branching/recombination reactions versus chain scission, resulting in PBSA structural changes, which would result in enhanced rheological and mechanical performance. This RP design would not require chemical additives or chain extenders, resulting in a cost-effective single-step, green strategy. Continuous twin screw extruders (TSE) are considered the more suitable equipment for RP because they provide good melt mixing and mass transfer among the different components, improving the homogeneity of chemical reactions during melt processing.^2,27,29^ However, before transferring a chemical reaction into a continuous melt processing in TSE, batch melt processing equipment, such as internal mixers, is more suitable to provide a preliminary study since it allows processing control over time, providing essential information for the appropriate design of a continuous RP process. In this study, the temperature, screw rotation speed, and residence time were studied to evaluate the influence of the processing parameters on the thermomechanical degradation of a commercial PBSA in an internal mixer. Furthermore, we elucidate the mechanisms of the degradative/reactive path of PBSA as a proof-of-concept but potentially applicable to other polyesters. Therefore, our work is intended to serve as a benchmark for using the RP as an effective tool for mutating bioplastic weaknesses into strengths.

## Experimental Section

### Materials

PTT MCC Biochem provided a commercial-grade biobased PBSA copolymer, identified as BioPBS FD92PM, which has a recommended processing temperature range of 130–150 °C and is certified for home composting by Vinçotte (European Union). Chloroform (ACS grade) was purchased from Sigma-Aldrich and used without any further treatment.

### Melt Processing Conditions

**Figure 1:**
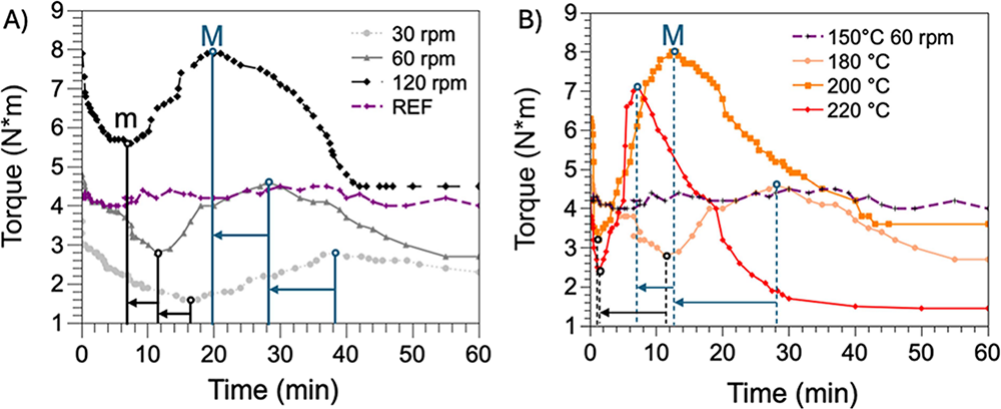
Time-dependent evolution of the torque recorded during PBSA processing in an internal mixer under different processing conditions. In particular, (A) at *T* = 180 °C and different screw speeds and (B) at 60 rpm and different processing temperatures.

The initial evaluation of reactive processing conditions was investigated on 40 g of PBSA, which was fed into a Brabender batch mixer operating at a predetermined temperature. The mixing speed was increased to the desired value within 10–15 s and kept constant for the test duration, which lasted for 60 min. Various temperatures and mixing speeds were used (Table 1, Figure 1). The test temperatures were 150, 180, 200, and 220 °C, and the mixing speeds were 30, 60, or 120 rpm (rpm). The experiments were carried out with the mixing bowl exposed to air with a relative humidity of 50–60%. During the mixing process, samples were taken from the mixing chamber according to established parameters and monitored throughout the torque curve. The samples collected from the mixer were immediately cooled in liquid nitrogen to prevent potential recombination and to inhibit any other reaction. Reactive melt processing (RP) of PBSAC1, PBSAC2, PBSAC3, and PBSAC4 was then carried out by variating the processing time, under constant processing parameters, selected after the initial evaluation by inline rheological analysis (*T* = 200 °C and *v* = 60 rpm) (Table 2).

**Table 1:** Main Melt Processing Parameters (Screw Speed and Temperature) Concerning the Online Rheological Assessment for the Different RP^a^

temperature control					shear control				
screw speed (rpm)	*T* (°C)	*t*_m_ (min)	*t*_M_ (min)	*t*_plateau_ (min)	screw speed (rpm)	*T* (°C)	*t*_m_ (min)	*t*_M_ (min)	*t*_plateau_ (min)
60	150	-	-	>1	60	150	-	-	>1
30	180	17	38	>35	60	180	12	38	>55
60	180	12	28	>50	60	200	2	12	>42
120	180	7	20	>40	60	220	2	6	>32

a In particular, when the melt processing parameters change, different times are needed for reaching the torque minimum (*t*_m_), maximum torque (*t*_M_), and torque at plateau (*t*_plateau_). (-) No torque minimum or maximum were detected for PBSA processed at 150 °C.

**Table 2:** Different Materials Were Produced in RP at 200 °C and Screw Speed of 60 rpm^a^

acronyms	processing time (min)	gel fraction (%)
PBSA150	12	-
PBSAC1	1	-
PBSAC2	2	-
PBSAC3	12	4.5
PBSAC4	45	2

a Acronyms, residential times, and measured gel fraction recovered after Soxhlet extraction from chloroform. (-) No gel fractions were detected.

### Rheological Characterization

The viscoelastic properties of the materials were evaluated via dynamic oscillatory rheometry in the molten state. A DHR-2 rheometer (TA Instruments) with a 25 mm parallel plate geometry was used for the tests at a temperature of 120 °C and a gap distance of 1.5–2 mm in a nitrogen environment. Amplitude stress and strain sweep test were first fulfilled, with an initial stress of 10 Pa and a strain of 1 × 10–5, up to a final strain of 2 at 0.628 rad/s. The complex modulus (G*), storage modulus (*G*′), and loss modulus (*G*″) were recorded as a function of stress (τ) and shear strain (γ), while a minor oscillatory amplitude strain (γ = γ_0_ sin(ωt)) was applied at a stress of 200 Pa and a strain of 0.1 rad. The moduli (G*, *G*′), complex viscosity (η*), and phase angle (δ) were then measured as a function of angular frequency (ω) between 0.01 and 100 rad/s.

### Size Exclusion Chromatography

The average molar mass and dispersity of three polyesters before and after degradation were analyzed using size exclusion chromatography (SEC). For this purpose, a Waters 1515 Isocratic HPLC pump (Milford, MA, USA) was employed, along with a Waters 1515 refractive index detector, temperature controller, and tungsten lamp at 35 °C. Chloroform (1.5 mg mL^–1^) was used as the eluent with a flow rate of 0.80 mL min^–1^ at 35 bar. A Waters Styragel HT column and different molecular masses of polystyrene ranging from 2000 to 10,000 g mol^–1^ were utilized as standards.

### Fourier Transform Infrared Spectroscopy

The materials were then analyzed using Fourier-transform infrared (FTIR) spectroscopy in the attenuated total reflection (ATR) mode. An FT-IR/NIR Spectrum 400 spectrophotometer (PerkinElmer, Waltham, MA, USA) was used to collect data in the 4000–400 cm^–1^ wavenumber range with a resolution of 4 cm^–1^. 32 scans were obtained from both films. The film was heated at 150 °C for 3 min to eliminate any thermal history and was then quickly transferred to a hot stage set at a prefixed temperature (60 °C) to ensure complete crystallization. The film was then used to collect its FTIR spectrum at room temperature. The spectra were normalized to the intensity of mode centered at 750 cm^–1^, which remained unchanged and deconvoluted using multipeak fitting analysis (Origin Lab 9.0 software).

### Nuclear Magnetic Resonance

^31^P NMR was obtained at ambient temperature on a Bruker Avance III HD 400 MHz instrument with a BBFO probe fitted with a Z-gradient coil for structural analysis. Data were processed through MestreNova (Mestrelab Research) with a shifted square sine-bell application window. Baseline and phase corrections were applied in both directions. The protocol for ^31^P NMR sample preparation and analysis was based on the one described by Argyropoulos in 1994.^30^

### Gel Content Assessment

The gel content of the PBSA sample was determined using the dissolution-extraction method according to ASTM D2765. The samples were dissolved in chloroform for 48 h, and the insoluble components were extracted and dried in a vacuum oven at 50 °C for 3 days to remove the chloroform. The amount of insoluble polymer residue was reported as the % gel content.

### Tensile Tests

The tensile performance on standard Dumbbells-shaped specimens with a thickness of 2 mm was evaluated according to the ASTM D638–14 standard, on a GB/T 1040.3–2006 on a CMT-4204 (SANS) tensile tester, with a crosshead speed of 3.5 mm/min (10% deformation rate). Before the tensile test, all specimens were conditioned for 24 h at 25 °C and 50% relative humidity. Subsequently, five dumbbell-shaped specimens of each sample were analyzed under the same conditions at room temperature.

### Wide Angle X-ray Diffractometry

The effects of degradation on the supramolecular structures of PBSA samples were studied using wide angle X-ray Diffractometry (WAXD). Measurements were taken on a Bruker D8 Advance diffractometer with a Cu Kα radiation source and a Bruker LynxEye 1D energy-dispersive detector. The diffractometer operated at 40 kV and 40 mA, with a Cu Kα source wavelength of λ = 1.54 Å. X-ray diffractograms were collected in the 2θ range of 5° to 60°, with a step of 0.05°. The crystallinity of the samples was calculated according to eq 1:
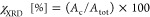
1where *A*_c_ is the area under the crystalline peaks of the spectra, while *A*_tot_ is the total area under the spectra between 2θ = 5° and 60°.

### Thermal Gravimetric Analysis

Thermal gravimetric analysis (TGA) 3+Starsystem (Mettler Toledo, Switzerland) was utilized to evaluate the thermal stability of the materials. A 3–5 mg sample was heated at 10 °C/min from 25 to 500 °C under a 60 mL/min nitrogen flow rate.

### Differential Scanning Calorimetry

The material’s thermal transitions and melting/fusion enthalpies were measured using a Mettler Toledo DSC2 calorimeter equipped with an HSS7 sensor and a TC-125MT intercooler. The endotherms were recorded when the temperature was raised from 25 to 160 °C, cooled back to −50 °C, and reheated to 150 °C, at a rate of 10 °C min^–1^ with a nitrogen flow of 60 mL min^–1^. The crystallization temperature (*T*_c_) from the cooling scan and melting temperature (*T*_m_) from the melting temperature during the second heating scan were taken as the peak value of the crystallization or melting enthalpy in triplicate measurements.

### Mechanical Recycling

Mechanical recycling was carried out by subjecting the samples to ten consecutive melt reprocessing cycles. For each cycle, the material was ground into pellets and melt processed in a corotating twin-screw microextruder (Xplore high torque micro compounder, MC 15 HT, The Netherlands) equipped with a microinjection molding unit (Xplore, The Netherlands). The conditions adopted for the extrusion were the following: *T* = 150 °C, speed = 60 rpm, and residence time = 5 min. Injection molding was performed under conditions of 150 °C and 6 bar injection pressure for 24 s.

## Results and Discussion

### Green Reactive Melt Processing Design

Since the thermomechanical degradation of PBSA exposed to relatively high temperatures and/or shear stresses^12^ can be monitored by viscosity decrease during melt processing,^12,31^ we investigated the influence of screw speed and temperature by an in-line torque assessment during the melt processing for 60 min of PBSA in an internal mixer (Figure 1A,B). Various screw speeds (30, 60, 120 rpm) were selected, maintaining a constant temperature of 180 °C (Figure 1A), while the effect of temperature was assessed by variating different temperatures (150, 180, 200, and 220 °C), keeping the screw speed constant at 60 rpm (Figure 1B). Melt mixing at 150 °C and 60 rpm (Figure 1A,B, purple dotted line) were selected as reference conditions demonstrating constant torque values over one hour of treatment. This result is consistent with the supplier’s recommendation to process the polymer below 160 °C to avoid thermal degradation.

For temperatures above 150 °C, the trend of torque proved to follow a different behavior depending on the processing conditions adopted. More in detail, all the torque curves recorded at temperatures higher than 160 °C showed a minimum (m) and a maximum (M) (Table 1), which was shifted to lower residence times by increasing either the processing temperature or the screw speed. After a maximum, a torque decrease indicates that PBSA melt viscosity decreases too, until a plateau is achieved. We hypothesized that the observed torque decrease could be ascribed to a reduction of favorable reaction kinetics for the development of RP. Such processing methods could lead to different PBSA architectures, resulting in varied rheological and mechanical properties. At the highest temperature tested, specifically 220 °C, a noticeable and rapid change in the torque profile was observed. After a steep increase in the first minutes, a swift decline in torque (i.e., viscosity) occurred, achieving a plateau after 30 min at the lowest recorded values (<1.5 N m). These results indicate that a mere 10 °C temperature increase has a more significant impact on degradation kinetics than doubling the screw speed from 60 to 120 rpm, thus intensifying the shear stresses. Furthermore, these findings suggest a similar kinetics for the initial chain scission, as indicated by the minimum torque being achieved after only 2 min of processing at both 200 and 220 °C. However, they also suggest faster kinetics for the hypothetical recombination and prevalent degradation occurring at 220 °C. With the objective of carrying out different RPs by varying the residential time, we identified four distinct intermediates that emerged during the melt processing at 200 °C and 60 rpm. Therefore, to study the effect of RP reaction time in these processing conditions, we prepared four separate batches of PBSA by stopping the processing after 1, 2, 12, and 45 min (Table 2). The resulting kinetic effect on RP was assessed by analyzing the structural, thermomechanical, and viscoelastic properties of the different reactively melt-processed PBSA and compared with the ones of commercial PBSA melt processed in nonreactive processing conditions.

### Structural Characterization

**Figure 2:**
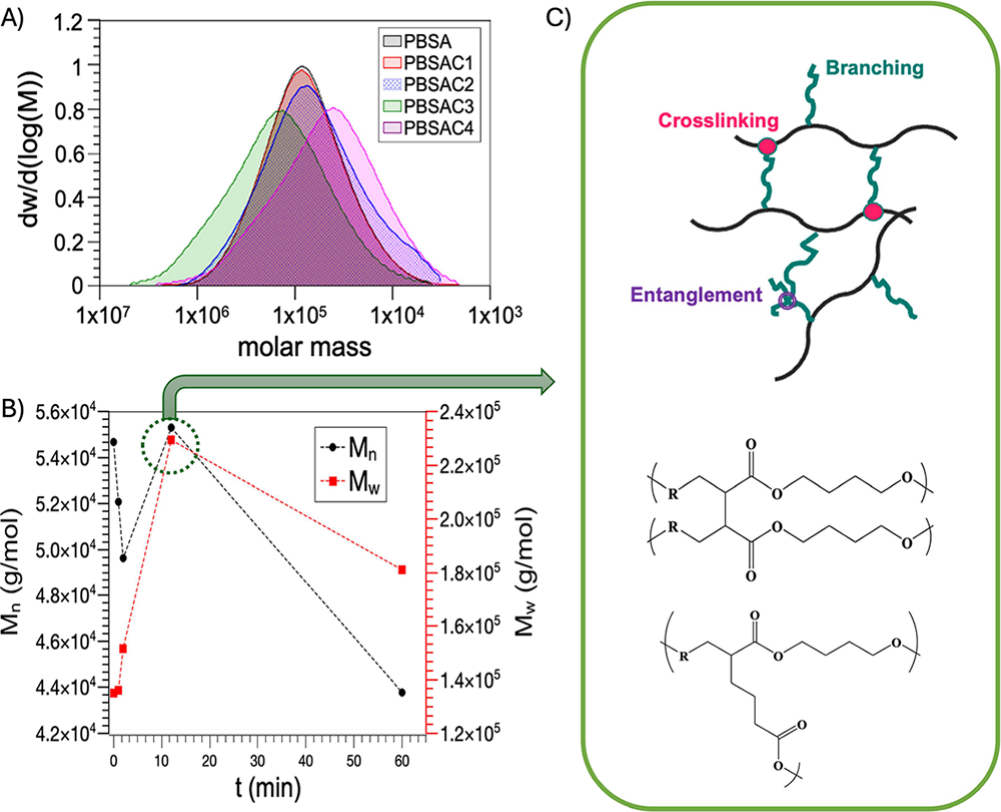
(A) Dispersity of unprocessed PBSA, and RP reacted PBSAC1, PBSAC2, PBSAC3, and PBSAC4 and (B) Number average molar mass (*M*_n_) and weight averaged molar mass (*M*_w_) as a function of processing time, measured by SEC. (C) scheme of branching/recombination.

SEC was carried out to gain structural information concerning the relative molar mass evolution as a function of the RP processing time. Dispersity, weight-average molar mass (*M*_w_), and number-average molar mass (*M*_n_) of the different samples were evaluated using RID (Figure 2) and UV–vis detectors (Table 3).

**Table 3:** Main Results of SEC Analysis Using the Refractive Index (RID) and UV–Vis Detectors for Unprocessed PBSA and RP Reacted Samples for Different Processing Times^a^

	refractive index detector (RID)					UV–vis detector	
samples	*M*_n_ (10^4^) g/mol	*M*_w_ (10^5^) g/mol	*M*_z_ (10^5^) g/mol	*M*_peak_ (10^4^) g/mol	*Đ* (*M*_w_/*M*_n_)	*M*_n_ (10^2^) g/mol	*M*_w_ (10^2^) g/mol
PBSA	5.46	1.34	2.87	8.64	2.4	-	-
PBSA150	5.28	1.37	3.03	8.30	2.6	-	-
PBSAC1	5.20	1.35	2.98	8.38	2.6	-	-
PBSAC2	4.96	1.51	3.67	8.05	3.2	7.14	8.53
PBSAC3	5.52	2.29	9.00	13.91	4.1	3.80	4.76
PBSAC4	4.37	1.81	5.64	4.41	4.1	9.71	10.62

a (-) No signals were detected.

**Figure 3:**
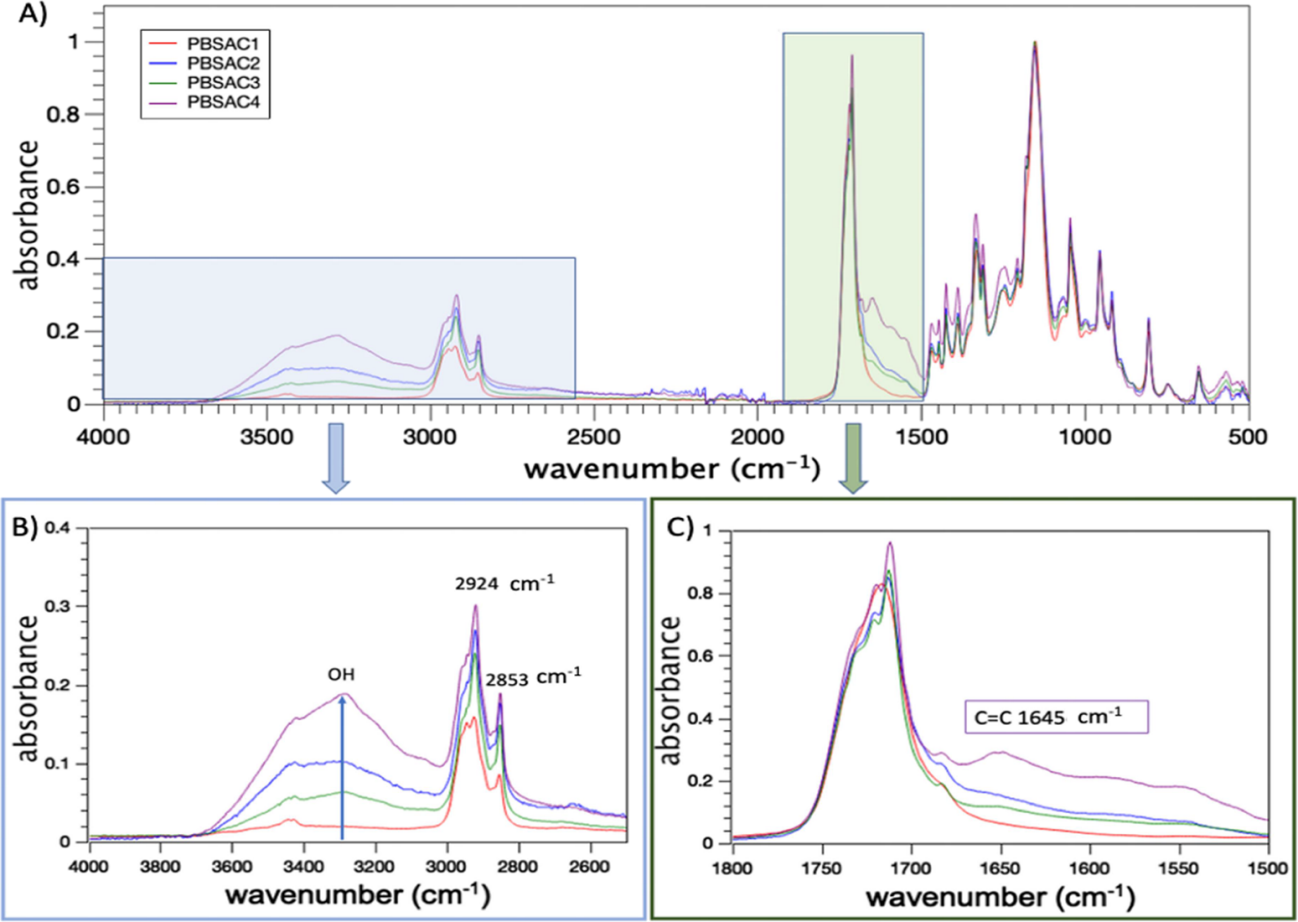
(A) FT-IR ATR spectra of PBSAC1, PBSAC2, PBSAC3, and PBSAC4 normalized to the intensity of mode centered at 750 cm^–1^. (B) Close-up view of the range 4000–2500 cm^–1^, showcasing the time-evolution of specific spectral features. (C) Close-up view of the range 1800–1500 cm^–1^.

The use of a UV–vis detector provides additional information on the molar mass of the sample compared to a RID, detecting the formation of unsaturated moieties. The results from the GPC analysis of the different PBSA samples are summarized in Table 3, and the normalized GPC curves are reported in Figure 2. GPC analysis confirms that RP, in the case of residence time lower than 2 min (PBSAC1), does not significantly alter the relative dispersity in comparison with the reference (PBSA150 processed at 150 °C for 12 min) and with unprocessed PBSA. PBSAC2 shows a broader dispersity toward lower molar mass, indicating that two min of RP resulted in a structural change of PBSA, inducing chain scission. The values obtained from the refractive index detector (RID) and plotted against the processing time demonstrate a progressive decrease in *M*_n_ (number average molar mass) starting from unprocessed PBSA, followed by PBSA150, PBSAC1, and PBSAC2, indicating a chain scission reaction that intensifies with higher temperatures and longer processing times (Figure 2B and Table 3). These findings suggest that a minute of RP treatment at the specified conditions or conventional melt processing at 150 °C for 12 min (PBSA150) does not significantly alter the structure of PBSA. However, extending the RP time to 12 min results in *M*_n_ values similar to unprocessed PBSA, indicating the prevalence of branching/recombination reactions over chain scissions. The evolution of *M*_n_ over time, as well as the *M*_w_ values, consistently increases with RP time up to 12 min (PBSAC1 < PBSAC2 < PBSAC3). Beyond this point, for longer RP durations, the *M*_w_ value starts to decrease (PBSAC4). Compared to unprocessed PBSA, the material subjected to 12 min of RP shows a notable 60% increase in *M*_w_ (weight average molar mass). Furthermore, for the material reactively mixed for 45 min, the molar mass decreased by approximately 20% compared to PBSAC3, yet it still maintained a molar mass of 25 and 33% higher than unprocessed PBSA and PBSAC1, respectively. Consequently, the polydispersity of the samples increases as the RP time extends, providing support for the hypothesized mechanism involving beta scission and branching/recombination during RP. The constant increase in polydispersity associated with prolonged processing times further supports the formation of branched macromolecules alongside shorter chains deriving from thermomechanical degradation. This hypothesis suggests that the observed structural changes indicate a progressively more branched structure, characterized by an initial stage dominated by scission, followed by a prevalent branching/recombination stage, Figure 2C, and finally, a third stage marked by predominant chain scission. Moreover, in PBSAC2, PBSAC3, and PBSAC4, macromolecular populations appear at the UV–vis detector, which were not detected in linear PBSA, PBSA150, or PBSAC1 (Table 3). These structures detected by UV–vis suggest the formation of macromolecules with unsaturated bonds during the processing. It is noteworthy that the *M*_n_ and *M*_w_ values observed under the UV–vis detector for PBSAC2 are approximately halved in PBSAC3 and then tripled in PBSAC4. Collectively, the findings from both the RID and UV–vis detectors indicate that the reactive processing process induces significant structural modifications in PBSAC3 and PBSAC4, leading to noticeable improvements in molar mass and polydispersity. FT-IR ATR spectroscopy was employed to track the structural changes in PBSA during processing (Figure 3A). The characteristic modes of PBSA were identified as follows: 1709 cm^–1^ (C=O stretching vibrations), 1151 cm^–1^ (C–O stretching mode), 1046 cm^–1^ (O(CH_2_)4O vibration), 955 cm^–1^ (C–O symmetric stretching mode), and 806 cm^–1^ (CH_2_ in OC(CH_2_)_2_CO in-plane bending mode)^32^ Previous studies have reported that the signals associated with the vibration of −CH groups (tertiary carbons) can be detected in the range of 2800–3000 cm^–1^ associated with CH_2_ stretching.^33^ On increasing RP processing time, the signal at 3300 cm^–1^ (Figure 3B), which corresponds to hydroxyl groups, intensifies. This intensification is directly linked to the formation of end-groups not previously present and the emergence of oxidized moieties. Furthermore, there is an observed increase of the band in the intensity at 1645, 952, and 914 cm^–1^ (associated with unsaturated groups)^9,18^ from PBSC2 to PBSC4 (Figure 3C). This indicates the existence of two competing thermomechanical oxidation pathways within the processing time ranges of 0–12 and 12–60 min. The former pathway appears to be dominant in the initial stages, leading to the generation of −OH moieties, C=C bonds, and oxidized compounds. The signal associated with stretching in the −C=C plane at 1645 cm^–1^ exhibits a notable dependency on processing time during reactive processing (Figure S1). The PBSAC2, PBSAC3, and PBSAC4 systems show a similar rising and falling trend for this signal, mirroring the behavior observed for the −OH groups. This consistency aligns with the results of molar mass measurements (*M*_w_ and *M*_n_) obtained from GPC analyses using a UV–vis detector, (Table 3). The intensity changes of the carbonyl band as the processing time progresses from PBSAC1 to PBSAC2, followed by a decline for PBSAC3 (Figure S1, yellow signal), can be attributed to recombination and branching reactions involving the aliphatic groups. The latter chain extinctions likely facilitate the formation of long chain branched structures, consistent with the observations reported in Figure 3A. Moreover, PBSAC4 shows a distinct enhancement of the band at 1645 cm^–1^, indicating that longer processing times could promote chain scission reactions and the subsequent formation of aliphatic complexes.^25,34−36^ The evolution of the terminal groups (hydroxyls and carboxylic acids) in the samples was evaluated by^37^P NMR (Figure S2).

**Scheme 1:**
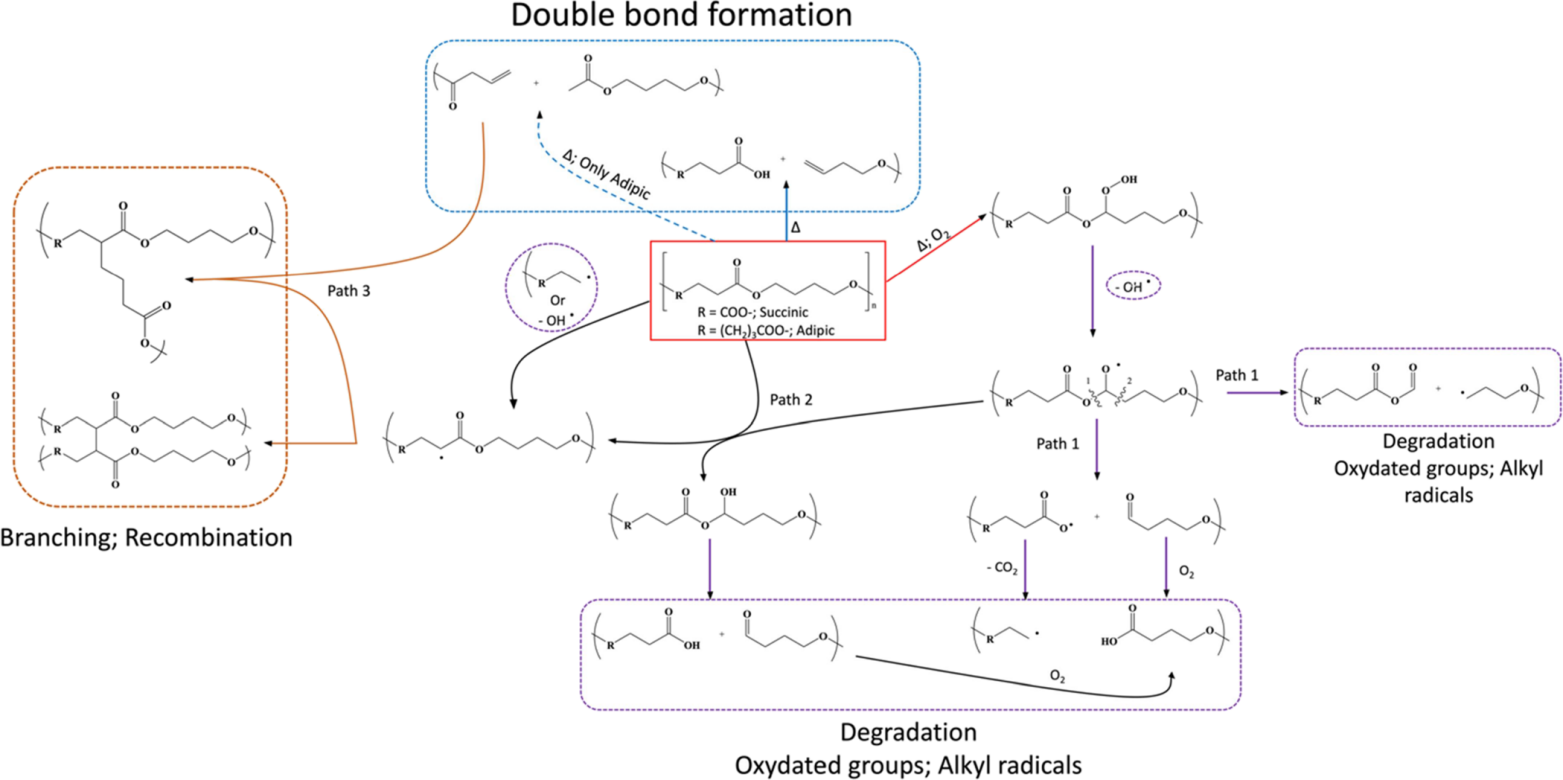
Closed-Loop Kinetic Scheme (CLS) Proposed for PBSA Modification during Green REx

The trend shows an increasing number of terminal groups with processing time, reaching a 25% increase in the level of PBSAC4 (Figure S2C). The carboxylic acids (+54%) give the most substantial contribution, while the −OH terminal groups remain roughly constant. However, considering the initial decrease in molar mass followed by a consistent increase (Table 3), the results suggest the formation and concomitant oxidation of the hydroxyls into carboxylic acids, followed by the creation of branching sites. FTIR spectroscopy, and ^31^PNMR analysis, along with selected literature references,^25−27,37,38^ suggested a panorama of the reaction mechanism, consisting of poly mechanical-oxidative closed-loop kinetic schemes (CLS) of PBSA (Scheme 1). Hydroperoxides, initially formed by polyoxidation, represent the starting sites that break down, producing radicals that may evolve according to different reaction pathways. This CLS scheme can be divided into the initiation of the chain scission (path 1), propagation of the radical chain fragmentation (path 2), recombination events such as branching and cross-linking (path 3), and depolymerization through cleavage of lateral groups and random chain splitting (path 4). In path 1, reactive groups, that is, −CO–CH–, −O–CO– form macroradicals such as alkyl and peroxide radicals. During the propagation (path 2), the macroradicals attack other macromolecules, thus, splitting long chains into shorter ones. Peroxide radicals may even evolve into alkyl by eliminating CO_2_. Autoxidation reactions occur in path 3, leading to the formation of oxygenated fragments, such as aliphatic carboxylic acids and alcohols. Path 4 is dominated by recombination events, which form products such as olefins, carboxylic acids, and ketones. The presence of branched macromolecules and 3D networks in the PBSA systems was confirmed through extraction using a Soxhlet apparatus. The results of extraction demonstrate that the PBSA systems can self-cross-link after 12 min of processing (Table 2), and cross-linked mass fraction can be tuned by varying the RP time. It is noteworthy that PBSAC3 exhibited the highest amount of the gel fraction. From these results, it can be concluded that the cross-linked gels in the PBSA systems exist as a network formed from a combination of intermolecular conjugates, polymer entanglements, and hydrogen bonds. These 3D networks result from the poly mechanical-oxidative closed-loop kinetic schemes (CLS) proposed for the reactive processing of the PBSA reaction mechanism, as depicted in Scheme 1.

### Rheological and Viscoelastic Properties

**Figure 4:**
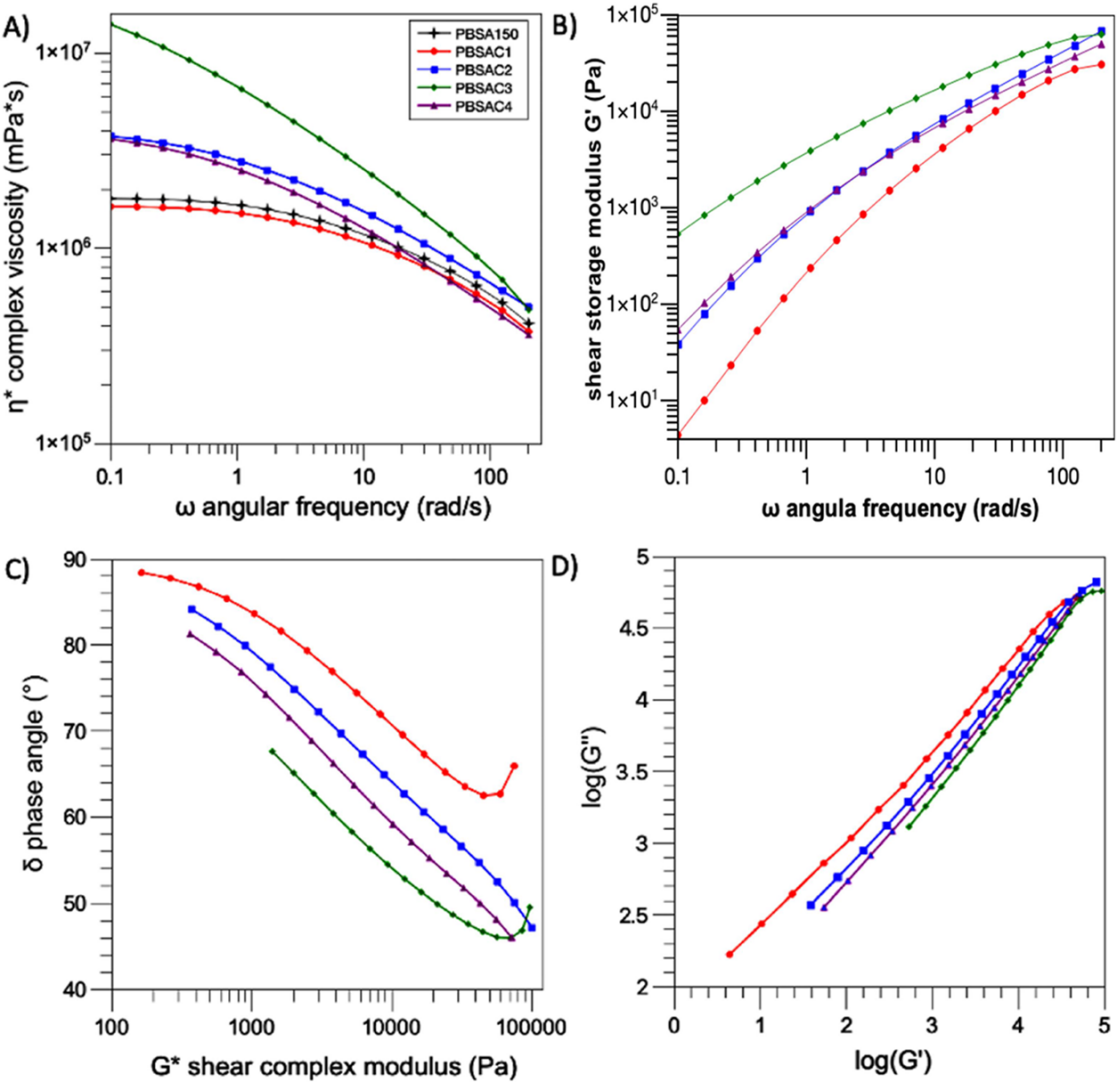
Rheological characterization of the samples processed at different times at 200 °C and 60 rpm: (A) Complex viscosity as a function of the angular frequency ω and (B) viscoelastic storage moduli, recorded during the frequency sweep tests in the molten state (*T* = 120 °C); (C) Van Gurp-Palmen plot, constructed by plotting the phase angle as a function of the complex shear modulus; and (D) Cole–Cole plots, obtained by diagramming log (*G*′) vs log (*G*″).

To investigate further the impact of different residence times on the PBSA architecture under reactive processing and conventional processing conditions (PBSA-150), rheological assessments were carried out (Figure 4). PBSA processed for 1 min at high temperature exhibited a rheological behavior similar to that of PBSA processed under conventional melt processing. In the low-frequency region, both these PBSAs displayed a Newtonian complex viscosity, followed by shear thinning starting at 1 rad/s (Figure 4A). This observation aligns with the similarity in molar mass between PBSA-150 and PBSAC1, indicating that reactive processing for only a minute did not induce significant effects. As the residence time in reactive processing increased, the rheological behavior of PBSAC2, PBSAC3, and PBSAC4 underwent noticeable changes, displaying more pronounced non-Newtonian complex viscosities. These alterations correspond to the torque values recorded during processing at the specified temperature and screw speed (Figure 1B) and corroborate the structural modifications occurring in PBSA during reactive melt mixing carried out for longer durations than a minute. The more prominent non-Newtonian behavior of PBSAC3, even at lower frequencies, can be attributed to an extended chain structure, leading to a higher number of entanglements and resulting in a higher complex viscosity.^38,39^ With prolonged RP, the complex viscosity decreases due to the lower molar mass of PBSAC4. However, despite having a lower molar mass, the complex viscosity of PBSAC4 remains higher than that of both PBSA150 and PBSAC1. This suggests the presence of a more branched molecular structure in PBSAC4. Among the different samples, PBSAC3 exhibits the largest shear storage moduli, exceeding PBSAC1 by two decades and PBSAC2 and PBSAC4 by one decade (Figure 4B). These changes in shear storage moduli agree with the observed increase in molar mass during RP between 2 and 12 min, indicating a higher level of entanglements and resulting in more elastic materials.^33,36,39^ The larger moduli of PBSAC4 can be attributed to its more branched structure in comparison to the moduli observed for linear PBSAC1. To gain a deeper understanding of the relationship between the rheological behavior and the macromolecular structure of PBSA, we utilized the Van Gurp-Palmen plot and the modified Cole–Cole plot. These analytical tools are instrumental in elucidating branching and chain scission, which are induced by chemical reactions occurring during melt processing.^40−42^ The Van Gurp-Palmen plot displays the phase angle δ as a function of the absolute value of the complex modulus G* for the reacted polyesters (Figure 4C). For PBSAC1, the phase angle initiates at 90° and gradually decreases with increasing complex modulus, indicating a typical viscoelastic fluid behavior with a prevalent dissipative liquid-like character.^42,43^ Conversely, PBSAC3 exhibits a phase angle δ that decreases from approximately 65° to below 45°, indicating a transition from liquidlike to solidlike viscoelastic behavior. This observation is consistent with the high molar mass detected and suggests a highly entangled polymer structure in the melt.^36^ The modified Cole–Cole plot illustrates the logarithmic relationship between the loss moduli *G*″ and the storage moduli *G*′ (Figure 4D). This analysis serves as a sensitive indicator of variations in macromolecular structure branching.^42,43^ The lower left region of the plot corresponds to Newtonian flow, where *G*″ is proportional to *G*′. The upper right region represents the viscoelastic properties in a rubbery plateau, indicating disentanglement of molecular chains. In the Cole–Cole plot of PBSC1, a broad transition across several decades of *G*′ moduli is observed. In contrast, the curves of PBSAC2, PBSAC4, and PBSAC3 show a progressive shift of *G*′ toward higher values and an increase in slope. These characteristics reflect a more elastic melt behavior, which can be attributed to increasing branching and the corresponding increase in gel fractions in cross-linked materials.^37−39,43^ To draw an analogy, the observed trend in RP-reacted PBSAs confirms an increasing branched structure from 2 to 12 min of RP, followed by chain scission in the case of PBSAC4. The material processed for an extended period (45 min) maintains a higher level of branching compared to linear PBSA and PBSAC1, indicating a greater degree of entanglement in the melt. However, the structural analysis confirms that the elasticity of PBSAC4 decreases compared to that of PBSAC3 due to the occurrence of chain scission. In the Cole–Cole plot curve of PBSAC3, the presence of the highest *G*′ values and slope indicates a higher level of entanglements in the melt, which can be attributed to branching. This observation is consistent with the previously discussed analysis of the molar mass.

### Mechanical Analysis

**Figure 5:**
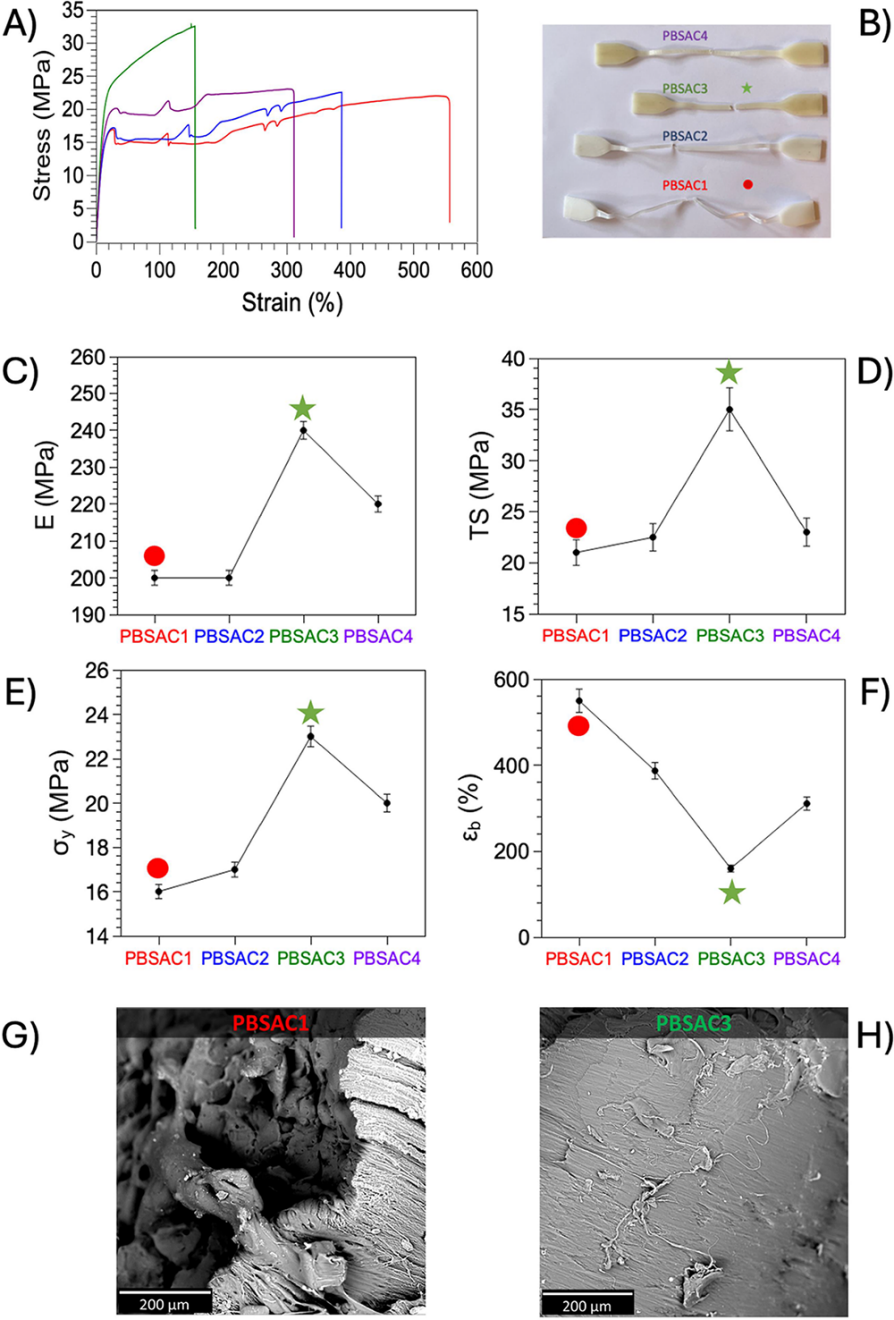
(A) Representative tensile stress–strain curves of PBSAC1, PBSAC2, PBSAC3, and PBSAC4; (B) digital photographs of Dumbbells’ specimens after tensile tests; (C) Young’s Moduli; (D) tensile strength; (E) yield stress; (F) elongation at break of the different materials; (G) cross sectional SEM micrographs of tensile fractured PBSAC1; and (H) PBSAC3.

Tensile testing was carried out to investigate the mechanical behavior of the materials and establish the relationship between the reactive processing of PBSA structures and their mechanical properties. The representative stress–strain curves (Figure 5A) reveal that all samples, except PBSAC3, exhibit the characteristic behavior of ductile materials, also displaying humps post yielding, likely arising from the formation and alignment (strain hardening), followed by failure of fibrils during stretching (Figure S3). In contrast, PBSAC3 does not show any rubbery plateauing and display directly after yielding massive strain hardening phenomena. This material showcases heightened stiffness, resistance, and decreased deformability compared with other systems, as evidenced by the visual inspection of the various specimens after tensile testing (digital photograph of Figure 5B). In fact, while the elastic moduli of PBSAC1 and PBSAC2 remained unchanged, the PBSAC3 system exhibited a significant 20% increase (Figure 5C). The stress–strain curve of PBSAC3 displayed the highest tensile strength (TS) and yield strength (Figure 5D,E), surpassing PBSAC1 by 86 and 62%, respectively. However, this increase in strength was accompanied by reduced ductility and deformation of the RP materials (Figure 5F and Table S1). It is suggested that the branching and cross-linking of polymer chains contributed to the formation of a structure that impeded the ordered folding of molecular chains. This structural alteration positively influenced the mechanical properties of the polymer, leading to enhanced intermolecular strength. The polymer branches, combined with the 3D cross-linked network, enhanced intermolecular forces by restricting the movement of molecular chains during deformation. This led to a strengthening and stiffening effect on the mechanical behavior of PBSAC3. However, this effect also resulted in a reduction in the strain at break observed for PBSAC3, as depicted in Figure 5B. Based on the aforementioned findings, the decrease in properties observed for the PBSAC4 system can be attributed to two conflicting effects: chain cleavage and cross-linking/branching events, which had opposing impacts on the elastic modulus and elongation at break (Figure 5C, F). Ultimately, the hyperbranching and gel network formed by the PBSAC3 polymer through RP established a stronger structure due to enhanced binding forces. This resulted in a distinct fracture mode compared to other samples, as verified by cross-sectional SEM micrographs of tensile fractured PBSAC2 (Figure 5G) and PBSAC3 (Figure 5H). PBSAC2 displayed cavitation and fibrillation phenomena, yielding a rough surface, whereas PBSAC3 exhibited a smoother surface.

### Thermal Analysis and Crystal Structure of RP PBSA System

**Figure 6:**
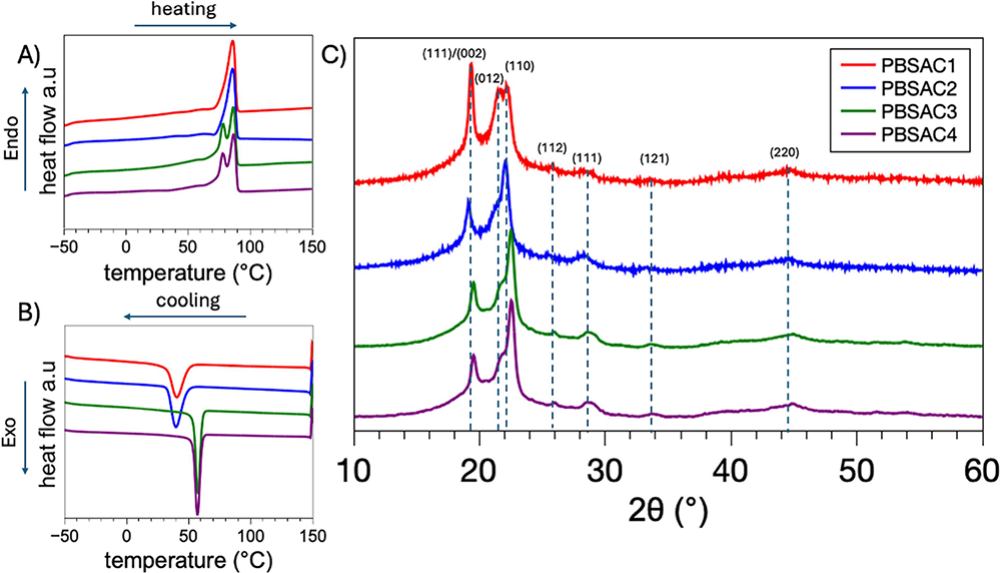
DSC analysis of PBSAC1, PBSAC2, PBSAC3 and PBSAC4: (A) scans collected during second heating, (B) cooling scans, and (C) WAXD patterns.

DSC analysis presented in Figure 6 provides crucial insights into the thermal and crystalline properties of PBSA and PBSA RP systems, including glass transition temperature (*T*_g_), melting temperature (*T*_m_), crystallization temperature (*T*_c_), and corresponding enthalpy changes (Δ*H*_m2_ and Δ*H*_c_) as shown in Table S2 and Figure 6A,B. The results indicate that RP has a significant impact on the thermal and crystalline properties of PBSA systems while not substantially altering the material’s thermal stability, as evidenced by the TGA analyses reported in Figure S4. Figure 6A reveals from the DSC analysis that the hyperbranched structure of PBSAC3 exhibits a lower enthalpy change *(*Δ*H*_m2_) compared to PBSAC1 and PBSAC2 (Table S2). Both PBSAC3 and PBSAC4 exhibit two melting temperatures (*T*_m1_ and *T*_m2_) at 78 and 87 °C, respectively, which is not observed in systems with low RP times (PBSAC1 and PBSAC2). Furthermore, during the cooling process (Figure 6B), it is noticeable that RP of PBSAC3 and PBSAC4 results in an increased *T*_c_ from 40 °C for PBSAC1 to 58 °C for PBSAC3. The shift in *T*_c_ can be attributed to increased branching and molar mass of the material, leading to a higher degree of chain entanglement and reduced chain mobility. Consequently, a greater amount of energy is required for the chains to rearrange and align during crystallization, resulting in a higher *T*_c_ and a decrease in the crystalline region’s perfection. The appearance of another melting peak *T*_m2_ in RP materials PBSAC3 and PBSAC4 can be ascribed to the presence of different populations of branched macromolecules with varying molar mass, which creates more amorphous regions in the material that melt at different temperatures.^10,44^ Furthermore, XRD analyses were performed to gain insight into the impact of RP-induced structural modifications on the organization of macromolecules within the crystalline lattice of the PBSA RP systems (Figure 6C). The XRD experimental data of PBSA reveals the presence of three diffraction peaks at 2θ = 19.4°, 21.7°, and 22.4°, corresponding to the (0202), (012), and (110) crystallographic planes of monoclinic PBSA, respectively.^38^ Analysis of the RP materials suggests that only the adipate comonomer undergoes structural alterations due to degradation. A multipeak fitting method was employed to distinguish the amorphous halo from the crystalline peaks and quantify the impact of degradation and branching on the supramolecular structures of the RP materials (Figure S5). Notably, significant structural changes are observed in the crystallographic planes associated with the adipate component with a marked reduction in the intensity of the (002) and (012) peaks. The degree of crystallinity was determined using eq 1, revealing a change in the PBSAC4 crystallinity, which exhibited an increased area under the peak assigned to crystalline region (Table S2). This increase in crystallinity for PBSAC4 is consistent with the observed decrease in the molar mass of PBSAC4.The increase in crystallinity observed in PBSAC4 following degradation is consistent with the thermooxidation reaction that occurred during the process. The amorphous regions of the polymer were initially broken down, leading to an increase in the crystallinity. The higher crystallinity observed in the PBSAC4 samples can be attributed to the fragmentation, depolymerization, and cleavage of lateral groups and random chain splitting described in step 4 of the CLS scheme. The comprehensive analysis of the DSC and XRD data establishes a strong correlation, highlighting that the RP has a profound influence on the thermal, crystalline, and supramolecular properties of the PBSA systems. This insight underscores the significance of comprehending the effects of RP on material properties, including their crystalline structures, in the pursuit of developing sustainable and efficient processes for their production and utilization.

### Mechanical Recycling of PBSA and RP PBSA

**Figure 7:**
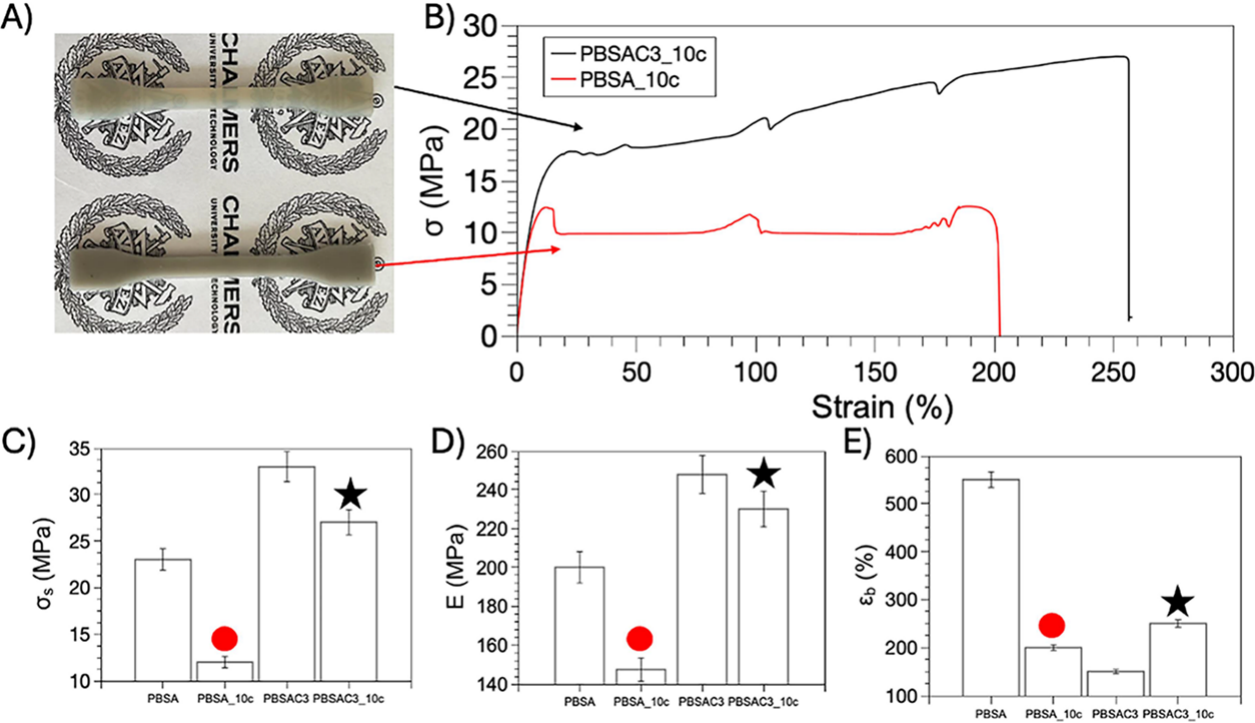
(A) Representative tensile stress–strain curves of PBSA neat and PBSAC3 after 10 cycles of mechanical recycling; (B) digital photographs showing the Dumbbells’ specimens appearance after 10 mechanical recycling processes; (C) Young’s Modulus; (D) tensile strength; and (E) elongation at break.

The recyclability of neat PBSA and PBSAC3 was investigated through multiple processing cycles (10 cycles at 150 °C and 60 rpm), involving microcompounding, injection molding, and pelletization (further details are reported in the experimental section). Figure 7A provides digital photographs of the dumbbell specimens for both samples after ten reprocessing cycles, labeled as PBSA_10c and PBSAC3_10c, respectively. The visual impact of mechanical recycling on the PBSA system is evident, as PBSA_10c exhibits a color change from white to gray. In contrast, PBSAC3–10c retains the same color as its unrecycled counterpart. Furthermore, Figure 7B shows notable differences in the stress–strain curves of the two recycled samples Figure 7A, with PBSAC3_10c displaying higher properties at the point of break compared to PBSA_10c. Figure 7C–E, respectively, report the mean values of elastic modulus, tensile strength, and elongation at break of the materials before and after recycling. Consistent with visual inspection, PBSA experiences a significant decline in all of its tensile properties after recycling. These findings indicate that the repeated processing cycles noticeably affect the material primarily due to the degradation of PBSA through chain scission during processing, leading to a significant decrease in the molar mass. This observation underscores the inherent weakness of the material, particularly its limitations in terms of recyclability. In contrast, PBSAC3 demonstrated good recyclability. After 10 extrusion processes, the RP system (PBSAC3_10c) only exhibits a slight reduction of approximately 6% in elastic modulus and 15% in tensile strength, while experiencing a 66% increase in elongation at break compared to the unrecycled sample. This seemingly surprising aspect could likely be attributed to a partial reduction in branching degree during the reprocessing steps, resulting in increased deformability.

## Conclusions

In this study, the effects of temperature, screw speed, and reactive processing on the thermomechanical degradative behavior, structural changes, and rheological and mechanical properties of PBSA were investigated. The goal was to design an environmentally friendly reactive processing process that exploits degradative compounds as reactive macromolecules, enhancing the mechanical properties and the recyclability of the material. The results demonstrate that PBSA is prone to degrade at high temperatures or shear stresses, leading to a more branched structure through notable chain scission and recombination reactions. Four reaction pathways were proposed for the reactive processing of PBSA, encompassing chain scission, chain fragmentation, recombination events, and depolymerization. By controlling the residence time during reactive melt processing, customized properties can be achieved, presenting opportunities for developing materials with the desired characteristics. Moreover, the mechanical properties of the reactive extruded PBSA (PBSAC3) exhibited significant improvement compared with those presented by PBSA conventionally extruded, with an 80% increase in strength and a 20% increase in stiffness. Additionally, PBSAC3 demonstrated excellent recyclability, allowing for up to 10 mechanical recycling cycles, surpassing the performance of the unreacted materials. These findings provide valuable insights into PBSA processing, enabling the development of enhanced processing techniques for this polymer and other polyesters. Furthermore, they unveil emerging potential applications for green reactive processing, broadening its scope and impact.

## Supporting Information Available

Deconvoluted carbonyl area for the RP materials evaluated by FTIR/ATR spectroscopy, evolution of the terminal groups (hydroxyls and carboxylic acids) for PBSAC1, PBSAC2, PBSAC3, and PBSAC4 evaluated by ^31^P NMR, digital photographs and SEM micrographs of PBSAC1 specimen providing further insights on the tensile behavior of materials, DSC, TGA, DTG, and WAXD patterns of PBSA RP samples with multipeak fitting to identify amorphous and crystalline signals (PDF)
