# Supplementary material for: Chemical-free Reactive Melt Processing of Biosourced Poly(butylene-succinate-adipate) for Improved Mechanical Properties and Recyclability
Source: ACS Appl Polym Mater. 2024 May 13;6(10):5866–77. doi: 10.1021/acsapm.4c00514 (PMC11129176; doi:10.1021/acsapm.4c00514)
Supplement: Supplementary file 1 — ap4c00514_si_001.pdf [file ap4c00514_si_001.pdf]

SUPPORTING INFORMATION

Chemical-free reactive melt processing of bio-sourced poly(butylene-succinate-adipate) for improved mechanical properties and recyclability

Michele Gammino[a], Claudio Gioia[b], Andrea Maio[a], Roberto Scaffaro[a]*, Giada Lo Re[c,d]*

a. Department of Engineering, University of Palermo, Viale delle Scienze, Ed. 6, 90128, Palermo, Italy.

b. Department of Physics, University of Trento, via Sommarive 14, 38123 Povo (TN), Italy .

c. Department of Industrial and Materials Science, Chalmers University of Technology, Rannvagen 2A, 41258 Gothenburg, Sweden.

d. Wallenberg Wood Science Centre, Kemigården 4, 41258 Gothenburg, Sweden.

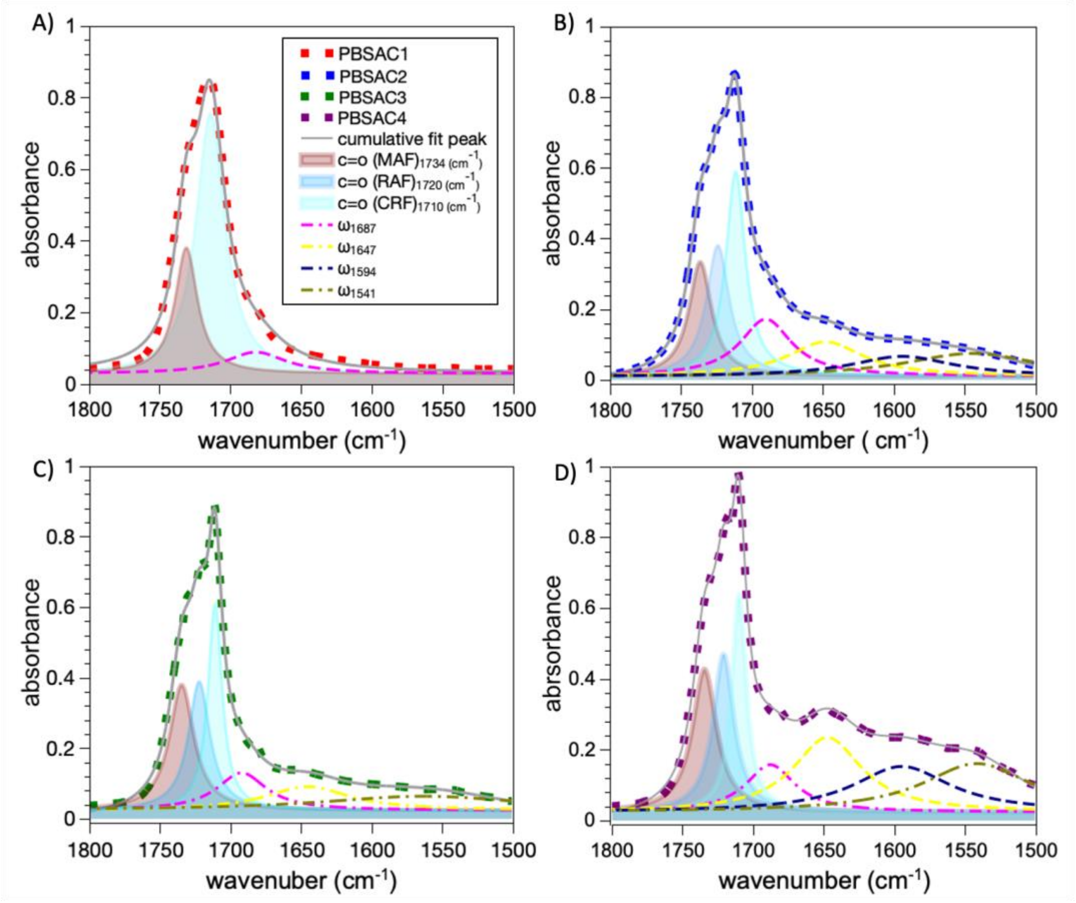

Figure S1. Deconvoluted carbonyl area for the REx materials PBSAC1(A), PBSAC2 (B), PBSAC3 (C) and PBSAC4 (D) samples.

Table S1. Mechanical properties of PBSA–lignin systems: Young's modulus (E), Yield strength (σ_y), Tensile strength (σ_b), Elongation at Break (ε_b).

Sample code	E (MPa)	σ_y (MPa)	σ_b (MPa)	ε_b (%)
PBSAC1	200 ± 3	16 ± 0.4	19 ± 0.5	550 ± 10
PBSAC2	200 ± 5	16.5 ± 1	23 ± 0.65	390 ± 23
PBSAC3	260 ± 2	24 ± 0.72	38 ± 1.2	150 ± 7
PBSAC4	220 ± 3	20 ± 1.3	23 ± 1.4	300 ± 13

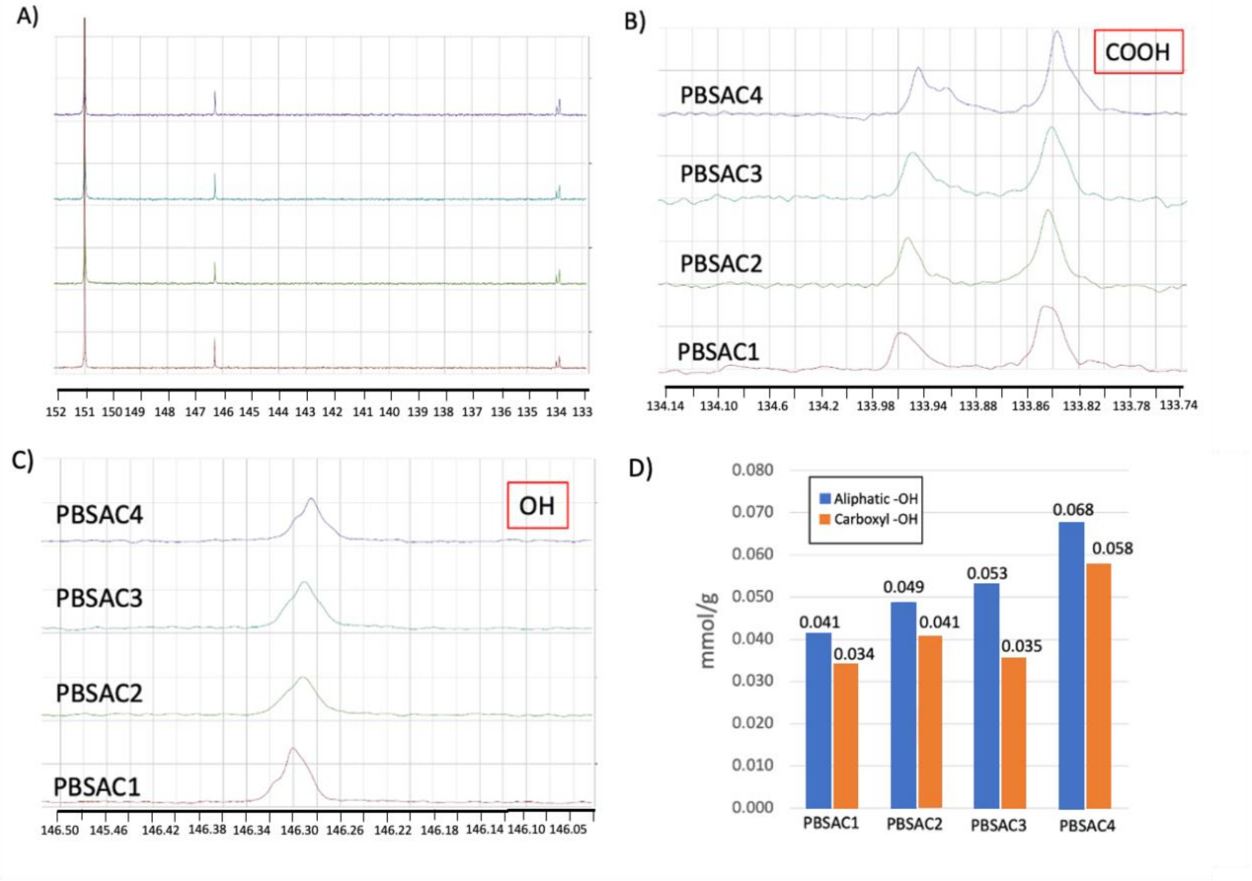

Figure S2. The evolution of the terminal groups (hydroxyls and carboxylic acids) for PBSAC1, PBSAC2, PBSAC3, and PBSAC4 evaluated by ^{31}P-NMR (A), inset in the region of carboxylic acids (B), in the hydroxyls groups (C), and their quantification (D).

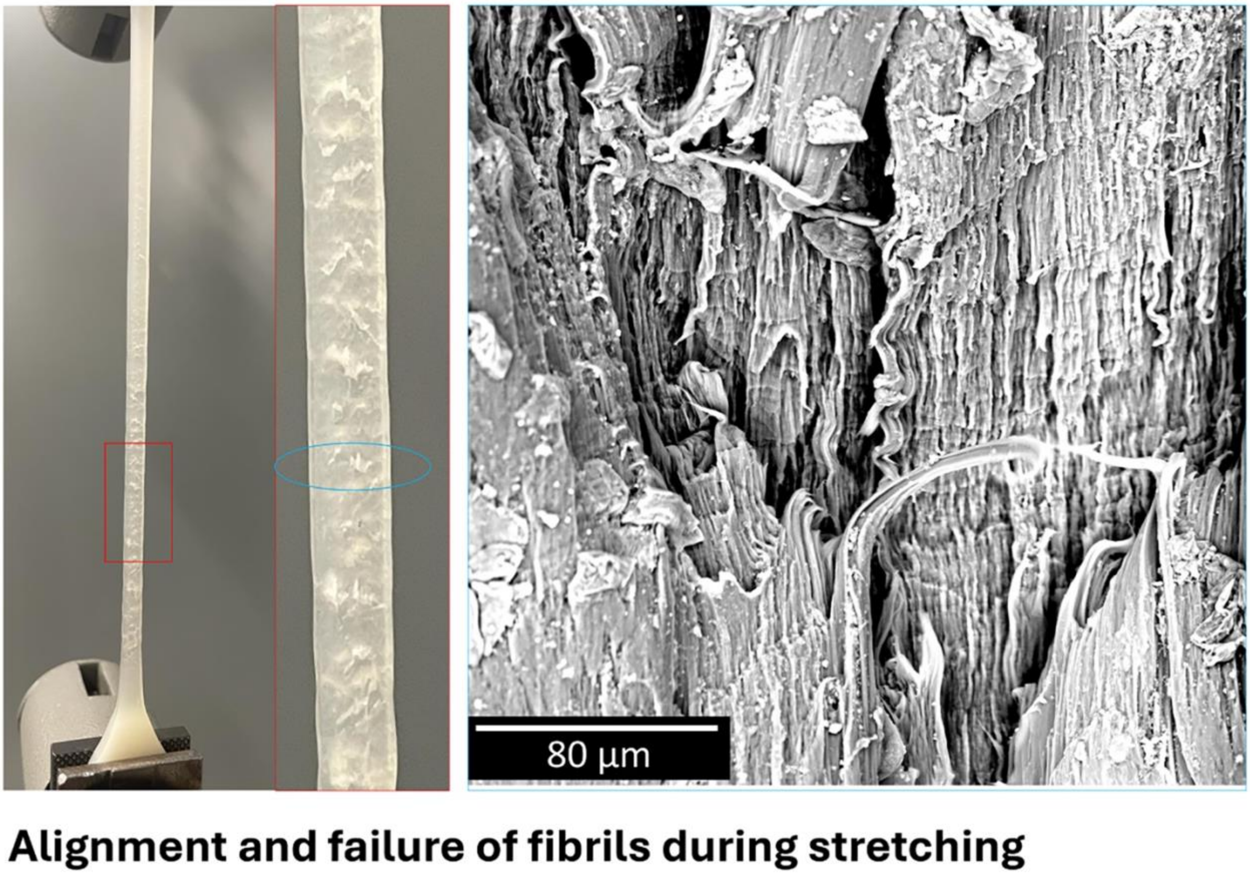

Figure S3. Digital photographs and SEM micrographs of PBSAC1 specimen providing further insights on the formation, alignment, and failure of fibrils during stretching, resulting in the humps detected in stress-strain curves of Fig. 5.

Table S2. DSC analysis of PBSAC1, PBSAC2, PBSAC3 and PBSAC4 sample.

Sample code	T_g (°C)	T_{m1} (°C)	T_{m2} (°C)	ΔH_{m2} (J*g^{-1})	T_c	ΔH_c (J*g^{-1})
PBSAC1	-44	-	86	35	40	37
PBSAC2	-44	-	87	33	40	36
PBSAC3	-46	78	87	32	58	36
PBSAC4	-45	78	87	43	57	40

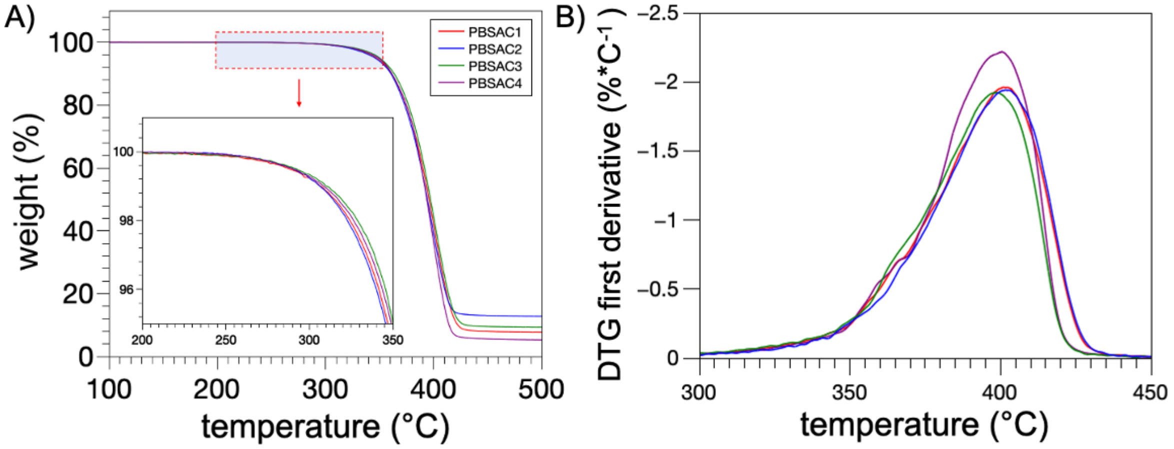

Figure S4. TGA (a) and DTG (b) thermogram of PBSAC1, PBSAC2, PBSAC3 and PBSAC4 samples.

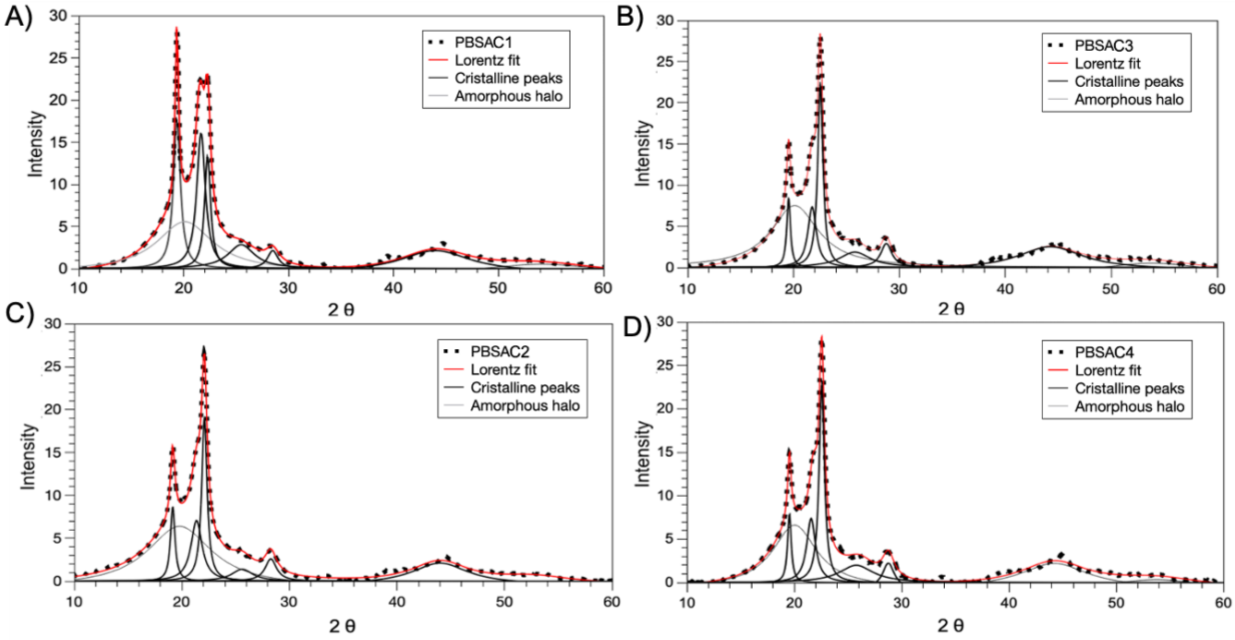

Figure S5. Wide Angle X-ray Diffractometry (WAXD) patterns of PBSA samples with multi-peak fitting to identify amorphous and crystalline signals.
